# Health Data for Research Through a Nationwide Privacy-Proof System in Belgium: Design and Implementation

**DOI:** 10.2196/11428

**Published:** 2018-11-19

**Authors:** Nicolas Delvaux, Bert Aertgeerts, Johan CH van Bussel, Geert Goderis, Bert Vaes, Mieke Vermandere

**Affiliations:** 1 Department of Public Health and Primary Care KU Leuven Leuven Belgium; 2 Sciensano Brussels Belgium

**Keywords:** electronic health records, health information exchange, health information interoperability, learning health systems, medical record linkage

## Abstract

**Background:**

Health data collected during routine care have important potential for reuse for other purposes, especially as part of a learning health system to advance the quality of care. Many sources of bias have been identified through the lifecycle of health data that could compromise the scientific integrity of these data. New data protection legislation requires research facilities to improve safety measures and, thus, ensure privacy.

**Objective:**

This study aims to address the question on how health data can be transferred from various sources and using multiple systems to a centralized platform, called Healthdata.be, while ensuring the accuracy, validity, safety, and privacy. In addition, the study demonstrates how these processes can be used in various research designs relevant for learning health systems.

**Methods:**

The Healthdata.be platform urges uniformity of the data registration at the primary source through the use of detailed clinical models. Data retrieval and transfer are organized through end-to-end encrypted electronic health channels, and data are encoded using token keys. In addition, patient identifiers are pseudonymized so that health data from the same patient collected across various sources can still be linked without compromising the deidentification.

**Results:**

The Healthdata.be platform currently collects data for >150 clinical registries in Belgium. We demonstrated how the data collection for the Belgian primary care morbidity register INTEGO is organized and how the Healthdata.be platform can be used for a cluster randomized trial.

**Conclusions:**

Collecting health data in various sources and linking these data to a single patient is a promising feature that can potentially address important concerns on the validity and quality of health data. Safe methods of data transfer without compromising privacy are capable of transporting these data from the primary data provider or clinician to a research facility. More research is required to demonstrate that these methods improve the quality of data collection, allowing researchers to rely on electronic health records as a valid source for scientific data.

## Introduction

More than a decade ago, the Institute of Medicine introduced the “learning health system” (LHS) in response to the challenges on how to generate and apply the best evidence to guide health care choices [1]. An important aim of the LHS is to enable the use of routinely collected health data for knowledge generation not only to ensure innovation in health care but also for the quality, safety, and value. In the LHS cycle, the analysis of routine health data creates new insights, which are then introduced back to health care providers through quality improvement tools such as clinical decision support, feedback, or audit reports. Even though the gold standard for measuring the effectiveness in health care has always been the randomized clinical trial, increasing attention is being given to registries and health data to contribute to evidence-based practice [2]. The use of health data recorded in the electronic health record (EHR) for research could help bridge the gap between evidence generated in controlled experiments and its application in daily clinical practice [3].

Even though the type of data recorded for research and the data stored in EHRs are similar, the use of health data poses some important problems. Concerns regarding the data quality and validity, completeness of data capture, and lack of interoperability have been identified as important barriers to the use of EHRs for clinical research [4]. Experiences from European and American efforts in the use or reuse of health data from EHRs have identified several important challenges [5,6]. Sources of bias included health care system bias, variations in EHR system functionalities and layout, and data extraction tools. These concerns have prompted opinions that the reuse of data for purposes other than that for which they were originally collected may be inappropriate [7]. Moreover, the European General Data Protection Regulation has further restricted the reuse of health data for research purposes [8,9]. Recent recommendations and guidance have proposed solutions to these concerns, but many challenges remain unanswered [10,11].

Perhaps, one of the most important challenges to the use of health data in clinical research is the persistent divide between clinicians (data providers) and researchers (data scientists) [12]. The incapacity to communicate between engineers and researchers, on the one hand, and clinicians, on the other hand, has resulted in disconnect between the world of research in medical informatics and the true problems in health care. In the evaluation and management of the health data for specific research questions, it is important that researchers understand the accuracy, comprehensiveness, retrievability, and specificity of health data recorded during routine care. On the other hand, clinicians need to be aware of the potential of the health data they record or manage and the implications that inconsistent or missing recordings may have on the reusability. Important facilitators to bridging this divide are enabling semantic interoperability, creating an environment of safe and reliable data transfer, ensuring privacy and security, and incentivizing valid and complete data capture [10]. In addition, experiences in the use of diverse sources of health data have led to a better understanding of data provenance (understanding of the authoritative source of a given data element of interest). For instance, if the measure of interest is whether a patient took a certain drug, then the best source for this outcome may not be the clinician’s order entry data but instead the nursing medication administration record. In this sense, data collection tools that allow the aggregation of health data across sources are important enablers of the LHS.

In Belgium, >150 clinical registries actively collect health data from multiple sources such as primary care facilities, laboratories, hospitals, and radiology centers. Moreover, there are multiple information systems or EHRs available for each of these sources. For example, for primary care practices alone, at least, 8 different EHRs are available. In 2012, the Scientific Institute of Public Health was charged with centralizing and improving these clinical registries as part of the national electronic health (eHealth) action plan in a new platform named Healthdata.be. The challenge for this task was to develop a system that allows the integration of data from diverse sources and collects them through multiple systems by clinicians during routine care, while ensuring the accuracy, validity, safety, and privacy of the data. This study addresses the following questions:

How can health data be transferred from various original sources of entry to a centralized platform for reuse and what efforts can be done to limit sources of bias?
How can health data within the LHS be used for various research designs?

This study will describe elements of the Healthdata.be project designed for data extraction, data transfer, and data processing. Subsequently, we will demonstrate how Healthdata.be was used in the INTEGO primary care morbidity registry [13] and in a cluster randomized trial in primary care [14].

## Methods

### Data Structure and Semantic Interoperability

**Figure 1:**
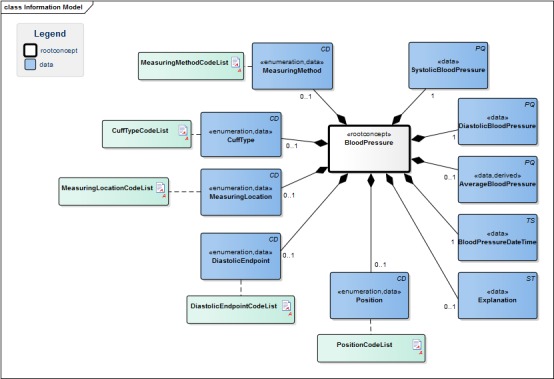
The detailed clinical model for the root concept blood pressure. CD: coded descriptor; PQ: physical quantity; TS: timestamp; ST: string (free) text. Source: https://www.healthdata.be/doc/cbb/index.php5/Be.en.hd.BloodPressure.

Health data are at the core of both EHRs and clinical research registries. However, to collect these data in a meaningful manner, these must have the same structure, use interoperable terminologies, and be documented using a detailed clinical model (DCM) [15,16]. DCMs provide detailed specifications of medical concepts in a given context and specify precisely the terminology to be used in terms of technical standards, reference models, and platforms [17,18]. They define all structured elements and attributes of a concept, including their relationships to the root concept, their data types, and the code lists that can be used. Where possible, code lists include internationally accepted coding such as logical observation identifiers names and codes (LOINC), Systematized Nomenclature of Medicine--Clinical Terms, International Classification of Diseases, international classification for primary care, etc. Figure 1 illustrates the DCM for the concept *blood pressure*, including all the associated data elements and their code lists as designed by the Netherlands Federation of University Medical Centres.

The content standardization of scientific data collections, using a DCM, contributes to the enhanced data quality and correct interpretation of data for research [19]. In addition, a substantial part of the information collected in the context of clinical registries is the continuity of care concepts (eg, diagnoses, medication, and laboratory test results), implying a certain overlap in content between different registries. Aligning all registries with DCMs, therefore, enables the harmonization across projects, allowing for the maximal reuse of existing data. As they are independent of technical aspects, such as message format, system, and network, DCMs can be considered technically neutral. Therefore, they can be used as a semantic layer between communication standards on the side of the primary source, and the parameters defined within different registries on the other side. Given the technological complexity and the variety of legacy systems, a stepwise approach for the registry standardization is proposed. In the first phase, existing registries are simply mapped to the DCMs. To maximize the degree of semantic interoperability, the registry structure can then be adjusted to comply with the logic defined in the DCMs. Registry variables that do not align with the DCM logic can still be completed using the manual interface. In the last phase, registries will be fully DCM-based, allowing the data provider to complete all registrations at the point of care.

Systems used by data providers are being urged to comply with these DCMs, and these elements are being included in local certification standards. When data providers or researchers are confronted with a concept for which no DCM exists, they can apply for the development of one to enable the automated provisioning of registries.

### Data Extraction

**Figure 2:**
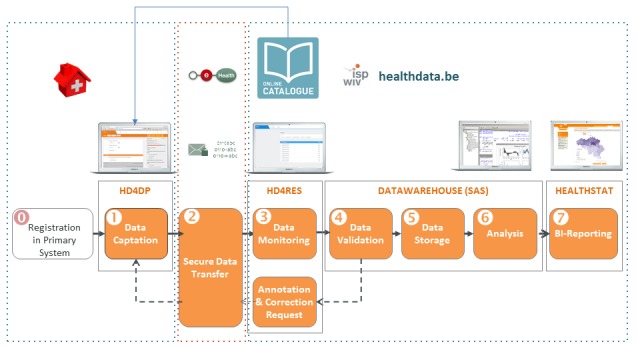
Data capture, data transfer, data encryption, and data reception through the Healthdata.be platform. HD4DP: Healthdata for data providers; HD4RES: Healthdata for research. Source: https://healthdata.wiv-isp.be/en/services.

When shaping the principles of LHSs, the Institute of Medicine reiterated the need to reflect on the burden data collection can be on health care professionals and the importance of limiting this burden to the issues most important to patient care and knowledge generation [20]. Healthdata.be has developed an electronic data capture (EDC) system called HD4DP (Healthdata for data providers), which enables data extraction at the primary source of clinicians. The logic defined in the DCMs is used in an application programming interface that serves as an interface between data providers’ primary source systems and the EDC. Supported input messages, based on health exchange standards, are mapped to the DCMs, upon which structured and coded information from primary source systems can be automatically prefilled in the EDC; this reflects the principle that health data should be recorded only once, and is expected to reduce the administrative burden for health professionals markedly. When data are entered through this data collection form (which can be Web-based but also installed locally), a comma separated value file is generated that can then be transferred on a patient-by-patient basis. Furthermore, data can be transferred in batch if the data provider’s system can generate this dataset with the variables automatically prefilled; this method for data capture is illustrated in Figure 2 in the box titled HD4DP.

### Data Transfer, Safety, and Privacy

The transfer of sensitive health data is challenging with regards to technicality, safety, and privacy. In Belgium, data transfer between health care professionals is organized through existing eHealth channels by an end-to-end (E2E) encryption [21]. For this data transfer, patient and provider identification is ensured through their unique social security identification number (SSIN), and data are encrypted with an algorithm that uses the SSIN of the sender and the receiver. This implies that only the sender or receiver can decrypt the message using their electronic identification card and pin code. The message is sent through an app called the eHealthBox [22], which can only be accessed using the same electronic identification card and pin code. Hence, this eHealthBox functions as a mailbox for encrypted health data. Institutions such as Healthdata.be also have an eHealthBox, which can be only accessed by individuals with the necessary security clearances. The transfer of health data from primary data providers to the Healthdata.be platform uses these channels of encrypted data transfer.

**Figure 3:**
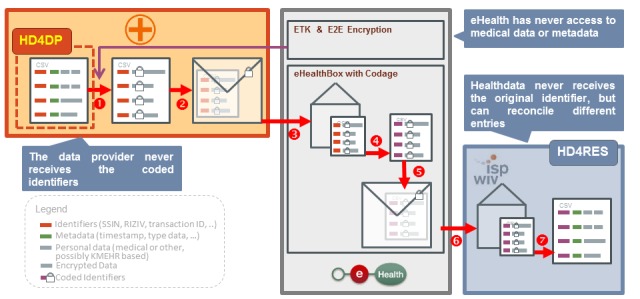
Illustration of the data encryption, coding and decryption steps; SSIN: social security identification number; HD4DP: Healthdata for data providers; HD4RES: Healthdata for researchers; CSV: comma separated value; ETK: eHealth token key; E2E: end-to-end. Source: https://healthdata.wiv-isp.be/en/services.

An important feature in enabling the linkage of health data extracted from different systems or settings is the ability to identify data from the same patient. When health data are being sent from one health care provider to another in the context of clinical care, the content of the message is encrypted, but the identity of the patient remains known. However, for research purposes, the content of the message is encrypted, but the identity of the patient must be blinded; this poses an important challenge when health data for a single patient are collected across sources. To enable this linkage without unblinding the coded data, an extra step is introduced in the data transfer. Where masking of identifiers is required, the national eHealth services act as a trusted third party and use an algorithm to pseudonymize this data element [23]. The algorithm used will always code the same patient identifier in the same way, ensuring that data from multiple sources from a single person will always be linkable but still coded. Healthdata.be is, therefore, not responsible for the coding and pseudonymization of sensitive data but uses existing eHealth services that have been technically tested and validated for this purpose. A critical moment in this chain of data transfer is the point where the data are received by the eHealth service and decrypted, before the identifier is coded. To ensure that a breach in this chain does not result in the loss of sensitive data, the whole message, except for the patient identifier, is encrypted the second time using an eHealth token key encryption known only by Healthdata.be [21]. This double encryption (eHealth token key encryption and E2E encryption) ensures safe and blinded data while allowing for the linkage of health data from multiple sources from a single person. All these processes, including the encryption and coding, happen automated and require no human input. Figure 3 illustrates each of the encryption, coding, transfer, and decryption steps. Step 1: data encryption (with exception of the identifiers); Step 2: encryption of whole message for data transfer through eHealth; Step 3: data transfer to trusted third party; Step 4: pseudonymization by trusted third party; Step 5: data encryption for transfer through eHealth; Step 6: data transfer to Healthdata.be eHealth:electronic health; Step 7: decryption of health data

Healthdata.be can process the data collection for very diverse specialties or research facilities in health care. To ensure that the requested data are in accordance with the research question or aim of the project, a thorough screening of the project is organized. Each project submits a research protocol, including a list of specific data variables being collected. An internal steering committee, an ethics committee associated with a research center, and the National Privacy Commission’s Sector Committee for eHealth review this submission. Only when all authorities have approved the project, can the data collection commence.

### Data Analysis

The Healthdata.be platform not only enables safe data transfer but also provides a secure environment for data handling and data analysis for research purposes. Coded data are received by the HD4RES (Healthdata for research) service, which shows the data as sent by the data provider. The interface of the HD4RES is almost identical to that of the HD4DP, except that identification details are coded. Upon arrival in the HD4RES, the data are not yet stored in the datawarehouse (DWH) of Healthdata.be. The DWH has 3 separate entities—the validation environment, the analysis environment, and the reporting environment—and uses SAS Enterprise Guide (SAS Institute Inc) to visualize and process the data. It is first stored in a validation table where data quality is controlled. Healthdata.be allows for semiautomated processes so that the validation of continuous data capture can be operationalized. Once validated, data are then promoted to the analysis environment of Healthdata.be. Access to the HD4RES and the separate environments of the DWH are secured through a 2-factor authentication and can be restricted depending on the needs of the researcher. Furthermore, data processing and reporting can be operationalized to accommodate a continuous data flow in ongoing registers.

### Feedback

**Figure 4:**
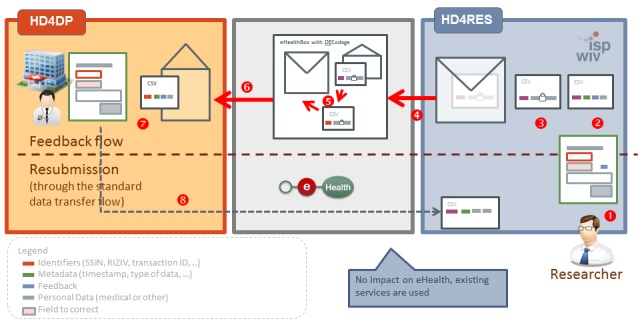
The illustration of the feedback loop in case of missing or erroneous data using the eHealth services. HD4DP: Healthdata for data providers; CSV: comma separated value; HD4RES: Healthdata for researchers. Source: https://healthdata.wiv-isp.be/en/services.

A pitfall to accepting data from various sources is the possibility of missing or erroneous data. Erroneous data can be prevented by introducing restricted possibilities, ranges, or syntaxes for the data transferred through the HD4DP. For example, validation rules that detect out-of-range data, missing data, or alphanumeric results for a numeric value can already prevent the transfer of these errors at the site of the data provider. However, it may still be possible that an aberrant value is transferred to the HD4RES that needs correction. To allow for this correction by the data provider, a feedback loop has been designed. This feedback loop uses the same channels and encryption methods for data transfer and includes decoding of the SSIN by the trusted third party of eHealth so that the primary data provider can identify the person for whom a corrected data variable is requested. Figure 4 presents this feedback loop.

## Results

### Case for INTEGO

INTEGO is a primary care morbidity registry, which was founded over 20 years ago [13]. The INTEGO registry has collected data from >100 general practitioners (GPs) and 400,000 patients since its start. Participating GPs all used Medidoc (Corilus, Aalter, Belgium) for documenting their clinical practice and were skilled in structured registration. Eligibility for participation in the INTEGO network required GPs to record on average, at least, one new diagnosis per patient per year, <5% noncoded diagnoses, and these 2 previous requirements should remain stable for, at least, 3 years. In 2017, further information technology development for Medidoc was terminated, and all its users were urged by the vendor to migrate to a new EHR software, called CareConnect (Corilus), a cloud-based EHR. This transition marked the moment to redesign the data collection for this registry, which had not changed since its start. To comply with the new General Data Protection Regulation legislation, the Healthdata.be platform was identified as a partner for this task.

INTEGO does not require any data collection besides that being collected for daily clinical practice. However, the quality of the collected data is expected to be of high quality. The data transferred to INTEGO not only include basic concepts, such as diagnoses or problems, procedures, prescriptions, laboratory tests, parameters, or vital signs and personal information, but also include intricate attributes such as longitudinal care for the same problem (problem-oriented medical registration), causal relationships between diagnoses and prescriptions, or the evolution of a health issue from a symptom into a diagnosis over time. Although many aspects of this registry were already described in existing DCMs, many of these attributes required additional coding and mapping to maintain their meaningfulness. The validity of the recorded data from the original INTEGO database has been studied through comparison with other existing continuous morbidity registries and found to be comparable [13,24]. There is little reason to assume that the data quality would change with the migration to the Healthdata.be platform, but continuous internal validity controls are ongoing.

The INTEGO procedures were approved by the KU Leuven Ethics Committee (nr. ML1723) and by the National Privacy Commission’s Sector Committee for eHealth (decision nr. 13.026 of March 19, 2013). The procedures to collect data by Healthdata.be were approved by the Belgian Privacy Commission on April 17, 2018.

To date, almost all GPs have migrated to CareConnect, and the first data export is being prepared and tested. On the one hand, a “core INTEGO” will be constructed, based on the original eligibility criteria to participate in the INTEGO network, to perform epidemiological research. On the other hand, an “extended INTEGO” will be constructed, without eligibility criteria to participate, to perform research on the quality of registration, quality of care, and impact of audit and feedback.

### Case for the Electronic Laboratory Medicine Ordering With Evidence-Based Order Sets in Primary Care Trial

The Electronic Laboratory Medicine ordering with evidence-based Order sets in primary care (ELMO) trial is a practical cluster randomized trial investigating the effects of decision support on the quantity and quality of laboratory test ordering behavior by GPs [14]. Data are collected from 3 separate sources on around 11,500 patients. Data on laboratory tests are provided by 3 private laboratories, each with a separate internal coding system for laboratory tests. The Belgian Terminology Center has introduced a national subset of LOINC codes, which are increasingly in use [25]. For the tests being investigated, mappings to LOINC codes were realized before the start of the trial to ensure interoperability. Data collection includes laboratory tests (type of test, value for test, and units), indications for laboratory test ordering, total cost of the test, identification of the physician, and identification of patients.

In addition, we collected patient-specific data directly from the GPs. GP investigators used several different EHR software for the registration of clinical practice. To ensure uniformity in the data collection, we designed a clinical report form (CRF), detailing the exact information we wished to extract from the EHR and which data would need to be added manually. To facilitate data extraction, we designed the CRF so that >60% of data would be automatically extracted from the EHR, meaning that these data already complied with one or more existing DCMs as defined by Healthdata.be and in use by most EHRs. The CRF was programmed and distributed to all EHRs through an app named Healthdata for Primary Care (HD4PrC), which is a tool that extracts the requested data directly from the EHR and populates the CRF with these data. Only data requests that could not be mapped to a DCM needed to be added manually. Examples of the requested patient-specific data were diagnoses or problems (including international classification for primary care codes and date of diagnosis), procedures performed or ordered, referrals to specialist care, pre- and posttest probabilities of disease, and diagnostic error. These data were then sent to the Healthdata.be platform through the described eHealth channels.

Finally, for a subset of patients, data were obtained directly from patients. A similar CRF was designed, which surveyed patients on data similar to the data requested from investigating GPs. Additional information on the socioeconomic status was requested. To ensure uniformity and avoid technical issues, the CRF was not sent directly to participating patients, but a telephonic interview was conducted by a research assistant who completed the CRF based on patients’ responses.

All these data were collected in separate SAS datasets on the Healthdata.be platform, which was accessible through a secured server. Access to various parts of the datasets was dependent on the role of the investigator, where data managers had access to the staging datasets, and statisticians had access to the analytics datasets. The chief investigator had access to all datasets and managed the authorizations of the entire team.

## Discussion

### Principal Findings

Healthdata.be has successfully connected a myriad of data providers on a centralized platform through a secure and private method of interoperable data transfer across settings and systems. This was done by enabling interoperable data collection, encrypted data transfer, and coded data collection while still allowing to connect data from the same patient collected from multiple sources through a system of pseudonymization. Healthdata.be has largely been able to bridge the disconnect between clinicians and researchers. In addition, Healthdata.be has been able to centralize >100 clinical registries governed by various research facilities, most of which are continuously collecting new data. A list of current registries being hosted on the Healthdata.be platform is available from the website (www.healthdata.be). Alongside the centralization of clinical registries, the platform can also be used in clinical trials or studies using routinely collected data at the point of care. Additional data, which are not defined through a DCM and specific to the trial or study, can be added manually to the data collection tool. These features make Healthdata.be an important facilitator of the LHS and help drive quality improvement in health care.

Facilitating access to reliable health data may be crucial to LHSs, but several situations have illustrated that there may be boundaries to this easier access. The Danish General Practice Database [26] was long considered an outstanding example of a clinical registry but was suspended because of concerns on privacy and security. Similarly, a large data-sharing database linking GP records with hospital data by the British National Health Service was terminated because of the lack of public confidence [27]. It does not appear to be a coincidence that public concerns on privacy coincide with patients’ increasing access to their own medical data [28]. In response to these concerns, Healthdata.be requires that all clinical registries or trials that wish to use its platform, receive approval from the National Privacy Commission’s Sector Committee for eHealth. This authority scrutinizes each app on security and privacy and determines whether the data collection is appropriate for the project. Projects that have not obtained approval are not operationalized within Healthdata.be.

### Limitations

An important limitation to the Healthdata.be platform is rooted in the decentralized data provision. Despite the efforts to standardize data collection using DCMs, variability in data collection at the point of care is inevitable. Even though DCMs may clearly define a clinical concept, there may still be variations in its use in documenting daily clinical practice. To be fully interoperable, DCMs must also be integrated into a conceptual model such as problem-oriented medical registration. These conceptual models not only include interoperable standards for individual concepts but also the relations between concepts. Moreover, even when a concept is well defined and documented within the same conceptual model, interrater differences persist; this is a feature that is common to the way the narrative of a patient is translated into an EHR documentation. Recording guidelines are required, and training on how to put these into practice is imperative, but harmonizing system designs and user interfaces may prove to be crucial. Many of the robust registries, such as the British General Practice Research Database [29] or the Dutch Sentinel General Practice Network [30], import their data from a minimal number of different systems to ensure validity. Many of the registries using the Healthdata.be service are also based on data from a single system, but it is also possible to use authentic sources for several data elements to ensure that the most accurate data are available. For instance, administrative data can be fetched from social security services in real time, ensuring that data, such as the date of birth, are correct. Similarly, like in the ELMO study, it is possible to access multiple systems and collect data where results are most likely to be accurate. In this sense, it seems logical to collect data on laboratory tests from the laboratory information system rather than from the GP EHR. However, no comparisons of the validity between registries collecting their data from a single source versus multiple sources have been done.

### Conclusions

The reuse of health data collected as part of routine clinical care can further research and improve health care. By ensuring semantic interoperability, safe data transfer, and trustworthy data handling, important sources of bias can be avoided. Concerns on data quality and validity can be addressed by collecting data from those sources where the data capture is bound to be most complete and linking these data from multiple sources through pseudonymization. Further research is required to assess whether these methods truly address concerns on the data quality. To date, patients have only limited access and cannot add or change health data in their own patient record. When these features become more widespread, it would be interesting to evaluate how this may influence the data quality and validity.

## Abbreviations

CRF: clinical report form
DCM: detailed clinical model
DWH: datawarehouse
EDC: electronic data capture
eHealth: electronic health
EHR: electronic health record
ELMO: Electronic Laboratory Medicine ordering with evidence-based Order sets in primary care
GP: general practitioner
LHS: learning health system
LOINC: logical observation identifiers names and codes
SSIN: social security identification number
